# Mrna vaccination under clozapine treatment

**DOI:** 10.1192/j.eurpsy.2022.675

**Published:** 2022-09-01

**Authors:** P. Argitis, A. Karampas, P. Mpouras, O. Pikou, F.-E. Meintanopoulou, S. Karavia, Z. Chaviaras

**Affiliations:** 1 General Hospital of Corfu, Psychiatric, Corfu, Greece; 2 General Hospital of Corfu, Dermatology, Corfu, Greece

**Keywords:** clozapine, covid

## Abstract

**Introduction:**

Common side effects are agranulocytosis and myocardiopathy. There are reports of myocardite related to m-rna vaccine for covid-19 (Pfizer-Bionteck), while the interactions with clozapine has not been yet studied.

**Objectives:**

The object of the study is to explore the safety of COVID-19 vaccination at patients treated with clozapine.

**Methods:**

We report a group of 27 patients from the psychiatric rehabilitation unit of the General Hospital of Corfu who were treated with clozapine and another group of 27 patients on different antipsychotic. Levels of clozapine were measured before the 1st vaccination and one month after the individuals were fully vaccinated, as well their COVID antibodies. For myocarditis detection we used CRP >1mg/dl and quadruplication of the troponin of reference.

**Results:**

No significant difference has been observed among the 2 groups in relation to antibody production, No difference has been detected between clozapine and nor-clozapine serum levels before and after vaccination. While there was no case of myocarditis or vein embolism noticed.

**Conclusions:**

It seems that patients treated with clozapine develop immune response to COVID-19 as individuals in any other anti-psychotic. No major side effects were reported at the two groups leading as to the conclusion that treatment with clozapine sould not be an obstacle for COVID-19 vaccination. Thus to the small number of patients in this study further research is needed.

**Disclosure:**

No significant relationships.

